# Metagenomic profiling dataset of bacterial communities of a drinking water supply system (DWSS) in the arid Namaqualand region, South Africa: Source (lower Orange River) to point-of-use (O'Kiep)

**DOI:** 10.1016/j.dib.2019.104135

**Published:** 2019-06-11

**Authors:** Innocentia G. Erdogan, Lukhanyo Mekuto, Seteno K.O. Ntwampe, Elvis Fosso-Kankeu, Frans B. Waanders

**Affiliations:** a Water Pollution Monitoring and Remediation Initiatives Research Group in the CoE C-based Fuels School of Chemical and Minerals Engineering, Faculty of Engineering, North-West University, Potchefstroom, South Africa; b Bioresource Engineering Research Group (*BioERG*), Cape Peninsula University of Technology, Cape Town, South Africa; c Department of Chemical Engineering, Cape Peninsula University of Technology, Cape Town, South Africa; d Department of Chemical Engineering, University of Johannesburg, Johannesburg, 2028, South Africa

**Keywords:** Drinking water supply system (DWSS), Metagenomics, O'Kiep, 16S rRNA gene

## Abstract

The metagenomic data presented herein contains the bacterial community profile of a drinking water supply system (DWSS) supplying O'Kiep, Namaqualand, South Africa. Representative samples from the source (Orange River) to the point of use (O'Kiep), through a 150km DWSS used for drinking water distribution were analysed for bacterial content. PCR amplification of the 16S rRNA V1–V3 regions was undertaken using oligonucleotide primers 27F and 518R subsequent to DNA extraction. The PCR amplicons were processed using the illumina^®^ reaction kits as per manufactures guidelines and sequenced using the illumina^®^ MiSeq-2000, by means of MiSeq V3 kit. The data obtained was processed using a bioinformatics QIIME software with a compatible fast nucleic acid (fna) file. The raw sequences were deposited at the National Centre of Biotechnology (NCBI) and the Sequence Read Archive (SRA) database, obtaining accession numbers for each species identified.

**Table:** Specification Table

Subject area	Drinking water supply system (DWSS), Biofilms, Microbial Ecology, Metagenomics
More specific subject area	Metagenomics
Type of data	Table
How data was acquired	Sequencing was performed on an illumina®MiSeq-2000, using MiSeq V3 (600 cycle) kits following procedures developed at Inqaba Biotech (Pretoria, South Africa) (www.inqababiotech.co.za)
Data format	Raw Data
Experimental factors	Metagenomic DNA was extracted from DWSS samples for sequencing.
Experimental features	Lower Orange River (source) [-28°90′8617″S, 18°20′7317″E] O'Kiep (point-of-use), South Africa [29°35′45″S, 17°52′51″E] Sample preparation: BioERG laboratory, Cape Town, South Africa [-33°93′0950″S, 18°43′3531″E]
Data source and location	DWSS, lower Orange River to O'Kiep, Namaqualand, South Africa
Data accessibility	The accession numbers of the sequences is publicly available on a public repository (http://hdl.handle.net/11189/6305) and are embedded within the supplementary materials.
Related research article	Richards, C.L., Broadaway, S.C., Eggers, M.J., Doyle, J., Pyle, B.H., Camper, A.K., Ford, T.E, 2018. Detection of Pathogenic and Non-pathogenic Bacteria in Drinking Water and Associated Biofilms on the Crow Reservation, Montana, USA. Microb Ecol 76: 52–63 [1] The research article that is associated with this article is still under construction.

**Table:** 

**Value of the data** • This data demonstrates the extent of bacterial contamination of a drinking water supply system in an arid region of Namaqualand, South Africa. • This data can be used to determine the role of the detected bacteria with the observed clinical abnormalities experienced by the O'Kiep community. • This data can also be used to develop mitigation techniques that will ensure that the drinking water is free of microbial contamination and suitable for drinking purposes.

## 1 Data

The presented data contains the microbial composition of a drinking water supply system (DWSS) for O'Kiep, Namaqualand, South Africa. Table 1 represents the bacterial composition of the source point at the lower Orange River while Table 2 shows the microbial composition of the treated water, distributed by a state owned agency responsible for water management activities in the region. Table 3 represents the microbial composition from a local municipal reservoir at O'Kiep storing the treated water from the water agency, which is further distributed to individual households in O'Kiep. Table 4, Table 5, Table 6, Table 7, Table 8, Table 9, Table 10 represents microbial composition at the point-of- use, i.e. households' tap.

**Table 1:** Bacterial community composition of the Orange River as identified by 16S rDNA amplicon gene sequencing.

Organism/HIT	%	Accession
Uncultured bacterium	70.31	gi|507147308|gb|KF037310.1|
Uncultured sphingomonas	2.39	gi|389547923|gb|JQ402851.1|
Uncultured pirellula	1.81	gi|192804906|emb|FM175708.1|
*Nocardioides* sp.	1.21	gi|119534933|gb|CP000509.1|
*Bacillus* sp.	1.18	gi|697883209|gb|KM205825.1|
Uncultured bosea	0.95	gi|238914939|gb|GQ129955.1|
Uncultured pseudonocardia	0.79	gi|56547765|gb|AY834333.1|
Uncultured frankineae	0.78	gi|192805020|emb|FM175822.1|
Uncultured actinobacterium	0.69	gi|110753753|gb|DQ828440.1|
*Proteobacterium*	0.69	gi|451916633|gb|KC450497.1|
*Pimelobacter simplex*	0.48	gi|723622094|gb|CP009896.1|
Uncultured sphingobacterium	0.41	gi|451919518|gb|KC453382.1|
*Proteobacterium*	0.40	gi|116687962|gb|AF114621.2|
Uncultured sphaerobacteraceae	0.26	gi|219906550|emb|AM935838.1|
Uncultured proteobacterium	0.25	gi|134020863|gb|EF019439.1|
Uncultured acidobacteria	0.25	gi|330340199|gb|JF521694.1|
Uncultured rhizobiales	0.18	gi|317448927|emb|FR695964.1|
Uncultured chloroflexi	0.18	gi|389547105|gb|JQ402033.1|
Uncultured micrococcaceae	0.16	gi|389547004|gb|JQ401932.1|
Uncultured anaerolineaceae	0.15	gi|389546919|gb|JQ401847.1|
Uncultured rubrobacter	0.14	gi|389546452|gb|JQ401380.1|
*Pantoea* sp.	0.14	gi|756794783|gb|KP326384.1|
*Variovorax paradoxus*	0.13	gi|239804838|gb|CP001636.1|

**Table 2:** Bacterial community composition of the treated water board agency reservoir as identified by 16S rDNA amplicon gene sequencing.

Organism/HIT	%	Accession
Uncultured bacterium	64.99	gi|206599296|gb|FJ206955.1|
Uncultured actinobacterium	6.70	gi|307092119|gb|HM480655.1|
*Actinophytocola xinjiangensis*	6.18	gi|636560203|ref|NR_116263.1|
*Myxococcus stipitatus*	4.36	gi|441484664|gb|CP004025.1|
*Mycobacterium neoaurum*	3.24	gi|674790876|gb|CP006936.2|
Uncultured anaerolineae	1.72	gi|219932282|emb|FM209128.1|
*Modestobacter marinus*	0.78	gi|388483940|emb|FO203431.1|
*Nocardioides* sp.	0.58	gi|119534933|gb|CP000509.1|
Uncultured acidobacteria	0.57	gi|341867197|gb|JN205269.1|
*Pimelobacter simplex*	0.54	gi|723622094|gb|CP009896.1|
*Proteobacterium*	0.42	gi|323709899|gb|HQ857672.1|
Uncultured proteobacterium	0.29	gi|110753316|gb|DQ828003.1|
Uncultured aquificae	0.28	gi|523452696|gb|KF183116.1|
Uncultured chloroflexi	0.27	gi|781796715|emb|LN797050.1|
Uncultured acidobacteriaceae	0.25	gi|192805191|emb|FM175993.1|
*Mycobacterium avium*	0.23	gi|701188573|gb|CP009614.1|
Uncultured microorganism	0.21	gi|478859630|gb|KC841593.1|
*Proteobacterium*	0.21	gi|825508410|gb|KR705964.1|
Uncultured planctomycete	0.16	gi|162287674|gb|EU299101.1|
Uncultured pseudonocardia	0.16	gi|56547765|gb|AY834333.1|
Uncultured rubrobacter	0.15	gi|389545690|gb|JQ400618.1|

**Table 3:** Bacterial community composition of the O'Kiep municipal reservoir as identified by 16S rDNA amplicon gene sequencing.

Organism/HIT	%	Accession
Uncultured bacterium	81.6	gi|399762709|gb|JX079102.1|
Uncultured verrucomicrobia	4.32	gi|325973802|emb|FR749796.1|
Uncultured pseudonocardia	1.61	gi|532020985|gb|KF150649.1|
*Nocardioides* sp.	0.88	gi|119534933|gb|CP000509.1|
Uncultured acidobacteria	0.87	gi|31789464|gb|AY281356.1|
*Natronomonas moolapensis*	0.67	gi|452081962|emb|HF582854.1|
Bradyrhizobium *sp.*	0.61	gi|146189981|emb|CU234118.1|
Uncultured rhizobiales	0.42	gi|630060146|gb|KJ191972.1|
*Desulfovibrio desulfuricans*	0.42	gi|219867585|gb|CP001358.1|
*Pimelobacter simplex*	0.36	gi|723622094|gb|CP009896.1|
*Conexibacter woesei*	0.35	gi|283945692|gb|CP001854.1|
*Sphingomonas* sp.	0.34	gi|918399443|emb|HF544321.2|
*Variovorax paradoxus*	0.33	gi|239799596|gb|CP001635.1|
*Modestobacter marinus*	0.30	gi|388483940|emb|FO203431.1|
Uncultured proteobacterium	0.27	gi|155008368|gb|EU052121.1|
Uncultured actinobacterium	0.25	gi|298231355|emb|FN811226.1|
*Mycobacterium smegmatis*	0.22	gi|433294648|gb|CP003078.1|
*Clavibacter michiganensis*	0.20	gi|147829108|emb|AM711867.1|
*Leptothrix cholodnii*	0.19	gi|170774137|gb|CP001013.1|
*Croceicoccus naphthovorans*	0.16	gi|831206920|gb|CP011770.1|
*Limnochorda pilosa*	0.15	gi|921142775|dbj|AP014924.1|
*Microvirga sp.*	0.14	gi|902761130|dbj|LC065182.1|
*Pandoraea apista*	0.13	gi|827413822|gb|CP011501.1|
Uncultured planctomycete	0.13	gi|197360261|gb|EU979049.1|

**Table 4:** Bacterial community composition of the household as identified by 16S rDNA amplicon gene sequencing.

Organism/HIT	%	Accession
Uncultured bacterium	81.6	gi|399762709|gb|JX079102.1|
Uncultured verrucomicrobia	4.32	gi|325973802|emb|FR749796.1|
Uncultured pseudonocardia	1.61	gi|532020985|gb|KF150649.1|
*Nocardioides* sp.	0.88	gi|119534933|gb|CP000509.1|
Uncultured acidobacteria	0.87	gi|31789464|gb|AY281356.1|
*Natronomonas moolapensis*	0.67	gi|452081962|emb|HF582854.1|
Bradyrhizobium *sp.*	0.61	gi|146189981|emb|CU234118.1|
Uncultured rhizobiales	0.42	gi|630060146|gb|KJ191972.1|
*Desulfovibrio desulfuricans*	0.42	gi|219867585|gb|CP001358.1|
*Pimelobacter simplex*	0.36	gi|723622094|gb|CP009896.1|
*Conexibacter woesei*	0.35	gi|283945692|gb|CP001854.1|
*Sphingomonas* sp.	0.34	gi|918399443|emb|HF544321.2|
*Variovorax paradoxus*	0.33	gi|239799596|gb|CP001635.1|
*Modestobacter marinus*	0.30	gi|388483940|emb|FO203431.1|
Uncultured proteobacterium	0.27	gi|155008368|gb|EU052121.1|
Uncultured actinobacterium	0.25	gi|298231355|emb|FN811226.1|
*Mycobacterium smegmatis*	0.22	gi|433294648|gb|CP003078.1|
*Clavibacter michiganensis*	0.20	gi|147829108|emb|AM711867.1|
*Leptothrix cholodnii*	0.19	gi|170774137|gb|CP001013.1|
*Croceicoccus naphthovorans*	0.16	gi|831206920|gb|CP011770.1|
*Limnochorda pilosa*	0.15	gi|921142775|dbj|AP014924.1|
*Microvirga sp.*	0.14	gi|902761130|dbj|LC065182.1|
*Pandoraea apista*	0.13	gi|827413822|gb|CP011501.1|
Uncultured planctomycete	0.13	gi|197360261|gb|EU979049.1|

## 2 Experimental design, materials and methods

### 2.1 Sample collection

The DWSS samples were obtained from a 100km long pipe system designed to deliver a flow of 18 ML/day. Freshwater is sourced from the lower Orange River by a regional water supply system to the nearby towns including O'Kiep which is located in the Northern Cape, Namaqualand region of South Africa [29°35′45″S, 17°52′51″E]. DWSS samples (n = 9) were collected in April 2017 from the source to the point-of-use, i.e. at numerous household taps, in non-transparent 500 mL sterile polyethylene bottles which were immediately placed on ice prior to transportation to the laboratory. A composite sample (n = 1) was initially collected from lower Orange River (Table 1). The second sample was composed of the treated water prior to distribution (n = 1) at the local water supply agency reservoir (Table 2). A similar composite sample (n = 1) from the local municipal reservoir (Table 3) and samples (n = 6) were randomly collected from households' taps (Table 4, Table 5, Table 6, Table 7, Table 8, Table 9, Table 10). All samples were handled according to the guidelines used for drinking water quality standard quantification [2], [3].

**Table 5:** Bacterial community composition of the household as identified by 16S rDNA amplicon gene sequencing.

Organism/HIT	%	Accession
Uncultured bacterium	68.84	gi|385762390|gb|JQ427676.1|
Uncultured modestobacter	10.87	gi|627529403|gb|KJ473576.1|
Uncultured pseudonocardia	2.99	gi|56547765|gb|AY834333.1|
Uncultured acidobacteria	1.82	gi|255669588|gb|GQ301073.1|
Uncultured micrococcineae	1.20	gi|192806380|emb|FM176888.1|
Uncultured actinobacterium	1.11	gi|197360258|gb|EU979046.1|
*Microbacterium* sp.	0.81	gi|166197412|dbj|AB376081.1|
Uncultured niastella	0.73	gi|429999989|gb|KC110902.1|
*Nocardioides* sp.	0.62	gi|119534933|gb|CP000509.1|
Uncultured beta proteobacterium	0.62	gi|451916627|gb|KC450491.1|
Uncultured actinomycete	0.48	gi|408830686|gb|JX507179.1|
*Pimelobacter simplex*	0.34	gi|723622094|gb|CP009896.1|
Uncultured proteobacterium	0.33	gi|781849781|emb|LN808336.1|
Uncultured planctomycete	0.31	gi|781829912|emb|LN803963.1|
*Kineococcus radiotolerans*	0.29	gi|196121877|gb|CP000750.2|
*Proteobacterium*	0.28	gi|238953279|emb|FM252918.1|
*Modestobacter marinus*	0.24	gi|388483940|emb|FO203431.1|
Uncultured streptomyces	0.23	gi|410699491|gb|JX576003.1|
Uncultured hyphomicrobium	0.23	gi|192805496|emb|FM176298.1|
Uncultured burkholderiales	0.21	gi|630060167|gb|KJ191993.1|
*Arthrobacter* sp.	0.18	gi|723606223|gb|CP007595.1|
*Rhodopseudomonas palustris*	0.14	gi|86570155|gb|CP000250.1|
Uncultured hyphomicrobiaceae	0.14	gi|389547438|gb|JQ402366.1|
*Ralstonia eutropha*	0.14	gi|113528459|emb|AM260480.1|

**Table 6:** Bacterial community composition of the household as identified by 16S rDNA amplicon gene sequencing.

Organism/HIT	%	Accession
Uncultured bacterium	81.15	gi|330372577|gb|JF340965.1|
Uncultured actinobacterium	3.87	gi|339646678|gb|JN037891.1|
Uncultured rhizobiales	2.37	gi|389546865|gb|JQ401793.1|
Uncultured acidobacteria	1.08	gi|430803015|gb|KC011124.1|
*Proteobacterium*	1.04	gi|18874511|gb|AF469355.1|
Uncultured planctomycete	0.80	gi|146430072|gb|EF220888.1|
*Nocardioides* sp.	0.79	gi|119534933|gb|CP000509.1|
Uncultured gemmatimonadetes	0.58	gi|151352239|gb|EF664948.1|
Uncultured anaerolineae	0.52	gi|219932282|emb|FM209128.1|
Uncultured actinomadura	0.48	gi|389546715|gb|JQ401643.1|
*Streptomyces* sp.	0.47	gi|822591927|gb|CP011492.1|
*Pimelobacter simplex*	0.44	gi|723622094|gb|CP009896.1|
Uncultured pirellula	0.33	gi|192804504|emb|FM175306.1|
*Proteobacterium*	0.29	gi|197360274|gb|EU979062.1|
Uncultured chloroflexi	0.27	gi|311336157|gb|HQ183884.1|
*Modestobacter marinus*	0.26	gi|388483940|emb|FO203431.1|
*Rhizobium* sp.	0.24	gi|584450787|emb|HG916852.1|
*Variovorax paradoxus*	0.20	gi|239799596|gb|CP001635.1|
Uncultured sphingomonas	0.20	gi|389547992|gb|JQ402920.1|
Uncultured frankineae	0.19	gi|192805020|emb|FM175822.1|
*Frankia alni*	0.15	gi|111147037|emb|CT573213.2|
Uncultured xiphinematobacteriaceae	0.14	gi|192806445|emb|FM176953.1|
Uncultured hyphomicrobiaceae	0.13	gi|166783119|gb|EU266779.1|
*Rhodopseudomonas palustris*	0.11	gi|39648490|emb|BX572598.1|

**Table 7:** Bacterial community composition of the household as identified by 16S rDNA amplicon gene sequencing.

Organism/HIT	%	Accession
Uncultured bacterium	77.81	gi|558611484|gb|KF711530.1|
*Proteobacterium*	1.68	gi|451914712|gb|KC448576.1|
Uncultured actinobacterium	0.98	gi|347438733|gb|JN178920.1|
*Alicyclobacillus acidocaldarius*	0.73	gi|339287872|gb|CP002902.1|
Proteobacterium	0.67	gi|294828896|gb|GU929355.1|
*Nocardioides* sp.	0.65	gi|119534933|gb|CP000509.1|
Uncultured rubrobacterales	0.58	gi|672229606|emb|HE861099.1|
Uncultured acidobacteria	0.56	gi|389545490|gb|JQ400418.1|
Uncultured anaerolineae	0.54	gi|219932282|emb|FM209128.1|
Uncultured proteobacterium	0.49	gi|110753058|gb|DQ827745.1|
Uncultured novosphingobium	0.45	gi|375271615|gb|JQ649064.1|
Uncultured cyanobacterium	0.35	gi|300679387|gb|HM439308.1|
*Pimelobacter simplex*	0.33	gi|723622094|gb|CP009896.1|
*Natronomonas moolapensis*	0.28	gi|452081962|emb|HF582854.1|
Uncultured janthinobacterium	0.27	gi|726973695|gb|KM391622.1|
Uncultured myxococcales	0.18	gi|389545327|gb|JQ400255.1|
*Microbacterium* sp.	0.18	gi|590121444|emb|HE716934.1|
Uncultured hyphomicrobiaceae	0.17	gi|166783147|gb|EU266807.1|
*Variovorax paradoxus*	0.17	gi|239799596|gb|CP001635.1|
Uncultured verrucomicrobia	0.16	gi|523452882|gb|KF183302.1|
*Conexibacter woesei*	0.15	gi|283945692|gb|CP001854.1|
Uncultured prokaryote	0.14	gi|283463150|gb|GU208299.1|
*Modestobacter marinus*	0.14	gi|388483940|emb|FO203431.1|
Uncultured planctomycete	0.12	gi|523452694|gb|KF183114.1|

**Table 8:** Bacterial community composition of the household as identified by 16S rDNA amplicon gene sequencing.

Organism/HIT	%	Accession
Uncultured bacterium	73.89	gi|134021494|gb|EF020070.1|
Uncultured acidobacteria	5.01	gi|325147373|gb|HQ597354.1|
*Pseudonocardia* sp.	3.34	gi|124488038|gb|EF216352.1|
Uncultured singulisphaera	3.25	gi|343787932|gb|JN367174.1|
Uncultured firmicutes	2.81	gi|392522374|gb|JX041802.1|
*Proteobacterium*	1.59	gi|451918460|gb|KC452324.1|
Uncultured actinobacterium	1.42	gi|110753103|gb|DQ827790.1|
Uncultured verrucomicrobiales	1.04	gi|192804575|emb|FM175377.1|
Uncultured balneimonas	1.01	gi|389548038|gb|JQ402966.1|
*Enterococcus hirae*	0.72	gi|94467694|gb|DQ467841.1|
*Plasticicumulans acidivorans*	0.45	gi|645320195|ref|NR_117458.1|
Uncultured proteobacterium	0.27	gi|781795286|emb|LN796725.1|
*Pseudonocardia dioxanivorans*	0.24	gi|444304041|ref|NR_074465.1|
*Frankia alni*	0.22	gi|111147037|emb|CT573213.2|
Uncultured planctomycete	0.19	gi|344050678|gb|JN409084.1|
*Rhodococcus sp.*	0.18	gi|909638169|emb|LN867321.1|
Uncultured earthworm	0.17	gi|25989809|gb|AY154543.1|
Uncultured carnobacterium	0.16	gi|319659383|gb|HM565028.1|
*Nocardioides* sp.	0.13	gi|119534933|gb|CP000509.1|
*Pimelobacter simplex*	0.12	gi|723622094|gb|CP009896.1|
*Sphingomonas wittichii*	0.12	gi|148498119|gb|CP000699.1|
Uncultured chloroflexi	0.12	gi|219896099|emb|AM934855.1|
*Microbacterium* sp.	0.11	gi|76252801|emb|AM051266.1|
*Actinomycetospora sp.*	0.10	gi|557126830|gb|KF600710.1|

**Table 9:** Bacterial community composition of the household as identified by 16S rDNA amplicon gene sequencing.

Organism/HIT	%	Accession
Uncultured bacterium	73.91	gi|399762838|gb|JX079231.1|
Uncultured actinobacterium	6.32	gi|781841436|emb|LN806491.1|
*Blastococcus saxobsidens*	3.71	gi|378781357|emb|FO117623.1|
Uncultured proteobacterium	2.31	gi|781835702|emb|LN805367.1|
*Methylocystis bryophila*	1.90	gi|384080409|emb|HE798551.1|
*Marmoricola* sp.	1.29	gi|384157059|gb|JQ419668.1|
Uncultured acidobacteria	1.02	gi|375271308|gb|JQ648757.1|
Uncultured rhizobiales	0.94	gi|389546865|gb|JQ401793.1|
*Proteobacterium*	0.71	gi|583826818|emb|HG917246.1|
*Microbacterium* sp.	0.67	gi|166197412|dbj|AB376081.1|
Uncultured anaerolineae	0.62	gi|523452566|gb|KF182986.1|
Uncultured pirellula	0.49	gi|545344262|gb|KF507494.1|
Uncultured planctomycete	0.48	gi|380838170|gb|JN868141.1|
Uncultured flavisolibacter	0.31	gi|396083910|gb|JX114425.1|
*Pelomonas* sp.	0.26	gi|530445182|gb|KC914556.1|
Uncultured solirubrobacterales	0.24	gi|389546277|gb|JQ401205.1|
Uncultured planctomycetaceae	0.23	gi|389546841|gb|JQ401769.1|
Uncultured xiphinematobacteriaceae	0.14	gi|192806445|emb|FM176953.1|
*Pimelobacter simplex*	0.14	gi|723622094|gb|CP009896.1|
*Nocardioides* sp.	0.14	gi|119534933|gb|CP000509.1|
Uncultured chloroflexus	0.14	gi|307564378|gb|HM241129.1|
Uncultured planctomycetes	0.12	gi|219906527|emb|AM935815.1|
Uncultured chloroflexi	0.12	gi|389547105|gb|JQ402033.1|
Uncultured verminephrobacter	0.10	gi|630060094|gb|KJ191920.1|

**Table 10:** Bacterial community composition of the household as identified by 16S rDNA amplicon gene sequencing.

Organism/HIT	%	Accession
Uncultured bacterium	76.2	gi|301246918|gb|HM710267.1|
Uncultured solirubrobacterales	4.04	gi|389545531|gb|JQ400459.1|
Uncultured alpha proteobacterium	3.25	gi|451914712|gb|KC448576.1|
Uncultured actinobacterium	2.70	gi|298231355|emb|FN811226.1|
Uncultured acidobacteria	1.31	gi|396083926|gb|JX114441.1|
Uncultured rhodospirillaceae	0.60	gi|83999434|emb|AM159371.1|
Uncultured arthrobacter	0.53	gi|389546219|gb|JQ401147.1|
*Pimelobacter simplex*	0.50	gi|723622094|gb|CP009896.1|
*Nocardioides sp.*	0.30	gi|119534933|gb|CP000509.1|
Uncultured proteobacterium	0.28	gi|134021577|gb|EF020153.1|
Uncultured acidobacterium	0.28	gi|386649463|gb|JQ825225.1|
Uncultured bacteroidetes	0.28	gi|149393241|gb|EF612369.1|
Uncultured sphingomonas	0.24	gi|46812524|gb|AY569282.1|
Uncultured chloroflexi	0.21	gi|313576414|gb|HQ397210.1|
Uncultured chitinophaga	0.20	gi|672229257|emb|HE860750.1|
*Proteobacterium*	0.33	gi|56547781|gb|AY834349.1|
*Novosphingobium pentaromativorans*	0.17	gi|698178797|gb|CP009291.1|
Uncultured microorganism	0.17	gi|529086744|gb|KF275220.1|
*Microbacterium* sp.	0.17	gi|914697494|gb|CP012299.1|
*Modestobacter marinus*	0.16	gi|388483940|emb|FO203431.1|
Uncultured planctomycete	0.12	gi|443301414|emb|HE613591.1|
Uncultured planctomycetaceae	0.11	gi|389547008|gb|JQ401936.1|
Uncultured xanthomonas	0.11	gi|82792029|gb|DQ279336.1|
*Brachybacterium faecium*	0.10	gi|256558041|gb|CP001643.1|

### 2.2 DNA extraction and sequencing

The samples were filtered through a 0.22-μm micropore cellulose membrane (Merckmillipore, USA) and the membrane was pre-washed with a sterile saline solution followed by the isolation of the genomic DNA using a PowerWater^®^ DNA isolation kit (MO BIO Laboratories, Canada) as per the manufacturer guidelines. The DNA purity and concentration were quantified using a microspectrophotometry (NanoDrop™ 2000/2000c Spectrophotometers Technologies, Wilmington, DE) and the DNA concentration ranged from 10.7 to 17.3 ng/μL.

The purified DNA was PCR amplified using the 16S rRNA forward bacterial primers 27F–16S-50-AGAGTTTGATCMTGGCT- CAG-‘3 and reverse primers 518R-16S-50-ATTACCGCGGCTGCTGG- ‘3 [4] that targeted the V1 and V3 regions of the 16S rRNA. The PCR amplicons were sent for sequencing at Inqaba Biotechnical Industries (Pretoria, South Africa), a commercial NGS service provider. Briefly, the PCR amplicons were gel purified, end repaired and illumina^®^ specific adapter sequence were ligated to each amplicon. Following quantification, the samples were individually indexed, followed by a purification step. Amplicons were then sequenced using the illumina^®^ MiSeq-2000, using a MiSeq V3 (600 cycle) kit. Generally, 20 Mb of the data (2 x 300 bp long paired end reads) [5] were produced for each sample. The Basic Local Alignment Search Tool (BLAST)-based data analyses was performed using an Inqaba Biotech (Pretoria, South Africa) in-house developed data analysis system. Overall, sequences were deposited in two databases, i.e. the National Centre of Biotechnology (NCBI) and the Sequence Read Archive (SRA) database, prior to the generation of accession numbers for individual bacterial species.
